# Supplementary material for: Trumpet is an operating system for simple and robust cell-free biocomputing
Source: Nat Commun. 2023 Apr 20;14:2257. doi: 10.1038/s41467-023-37752-x (PMC10119096; doi:10.1038/s41467-023-37752-x)

Supplementary information for

Trumpet is an operating system for simple and robust cell-free biocomputing

Judee A. Sharon[1], Chelsea Dasrath[1], Aiden Fujiwara[2], Alessandro Synder[2], Mace Blank[1], Sam O'Brien[2], Lauren M. Aufdembrink[1], Aaron E. Engelhart[1], Katarzyna P. Adamala[1]*

[1] Department of Genetics, Cellular Biology, and Development, University of Minnesota, Twin Cities, Minneapolis, MN

[2] Department of Computer Science, University of Minnesota, Twin Cities, Minneapolis, MN

*Corresponding author: kadamala@umn.edu

SI Figure 1: (a) An example of a 1.5% DNA agarose gel showing that the restriction enzyme, PvuII, can digest a template in version 1 of aHOT 7.9. Each experiment was done three times independently.

(b) The un-annotated raw image of the gel from (a).

a.

Lane	Sample
1	Loading Dye
2	RNA Dye
3	v1aHOT7.9 + gate template 2330 + positive control 2331 = digest
4	v1aHOT7.9 + gate template 2330 + inputs 2334 + 2335= digest
5	v1aHOT7.9 + gate template 2330 +input 2334 = no digest
6	v1aHOT7.9 + gate template 2330 +input 2335 = no digest
7	v1aHOT7.9 + gate template only 2330 = no digest
8	NEB OneTaqStd Buffer 5x + gate template 2330 + positive control 2331 = digest
9	NEB OneTaqStd Buffer 5x + gate template 2330 + inputs 2334 + 2335= digest
10	NEB OneTaqStd Buffer 5x + gate template 2330 +input 2334 = no digest
11	NEB OneTaqStd Buffer 5x + gate template 2330 +input 2335 = no digest
12	NEB OneTaqStd Buffer 5x + gate template only 2330 = no digest
13	NEB Restriction Buffer 3.1 + gate template 2330 + inputs 2334 + 2335= digest
14	NEB Restriction Buffer 3.1 + gate template 2330 +input 2334 = no digest
15	NEB Restriction Buffer 3.1 + gate template 2330 +input 2335 = no digest
16	NEB Restriction Buffer 3.1 + gate template only 2330 = no digest
17	NEB Restriction Buffer 3.1 + gate template 2330 + positive control 2331 = digest
18	NEB CutSmart Buffer + gate template 2330 + positive control 2331 = digest
19	NEB CutSmart Buffer + gate template 2330 + inputs 2334 + 2335= digest
20	NEB CutSmart Buffer + gate template 2330 +input 2334 = no digest
21	NEB CutSmart Buffer + gate template 2330 +input 2335 = no digest
22	NEB CutSmart Buffer + gate template only 2330 = no digest

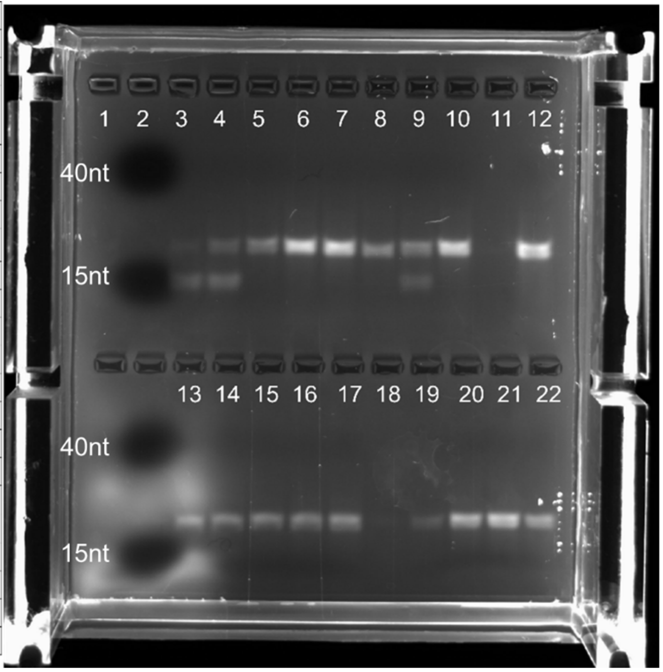

b.

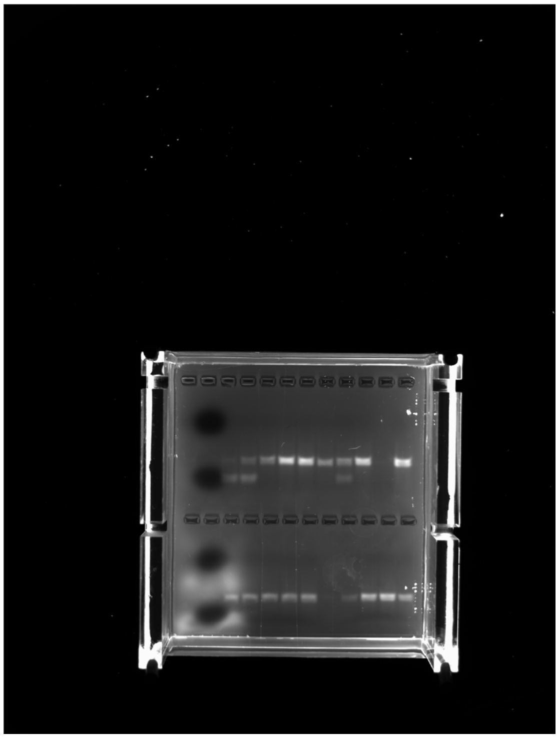

SI Figure 2: Four restriction enzymes were successful in digesting gate templates in aHOT 7.9 and cell free transcription shows that we can use fluorescent RNA aptamers as readout. While these early experiments were conducted with n=1 sample, all following experiments for this study were conducted with a minimum of n=3 samples. Apart from the restriction enzyme-specific gate samples, all experiments included cell free transcription controls (vBroccoli which was the Broccoli aptamer sequence alone) and a fluorescence standard to standardize between spectrophotometer readings (Fluorescein).

a.

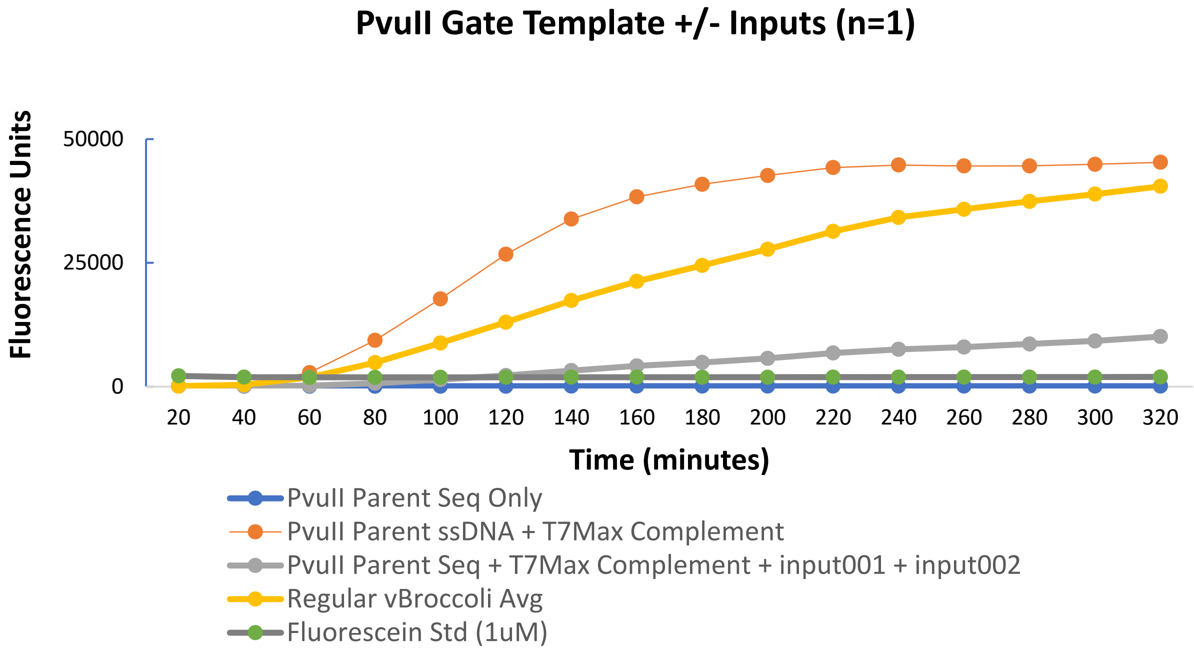

b.

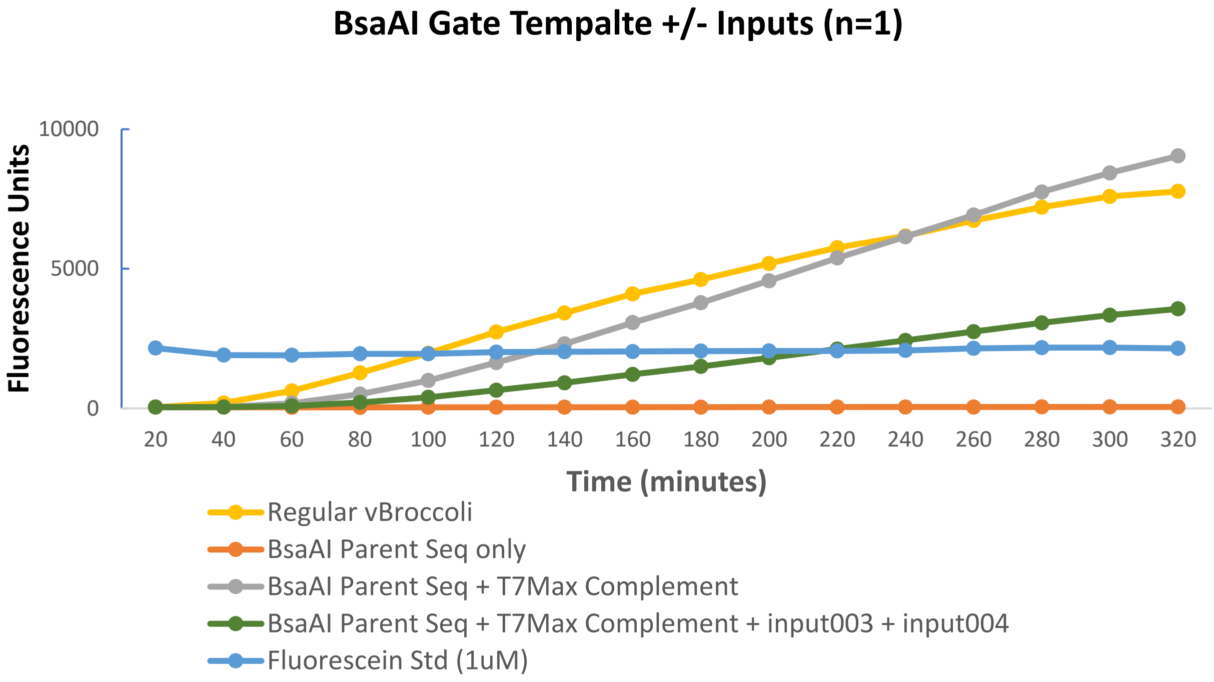

c.

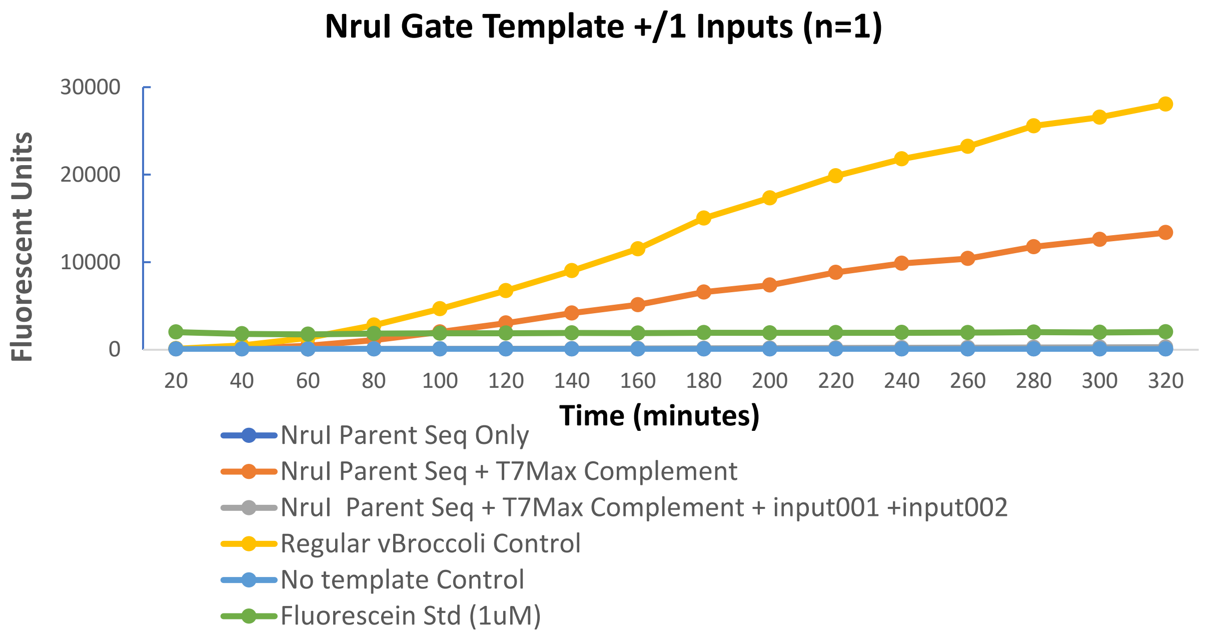
NruI Gate Template +/1 Inputs (n=1)
Fluorescent Units
30000
20000
10000
0
20 40 60 80 100 120 140 160 180 200 220 240 260 280 300 320
Time (minutes)
NruI Parent Seq Only
NruI Parent Seq + T7Max Complement
NruI Parent Seq + T7Max Complement + input001 +input002
Regular vBroccoli Control
No template Control
Fluorescein Std (1uM)

d.

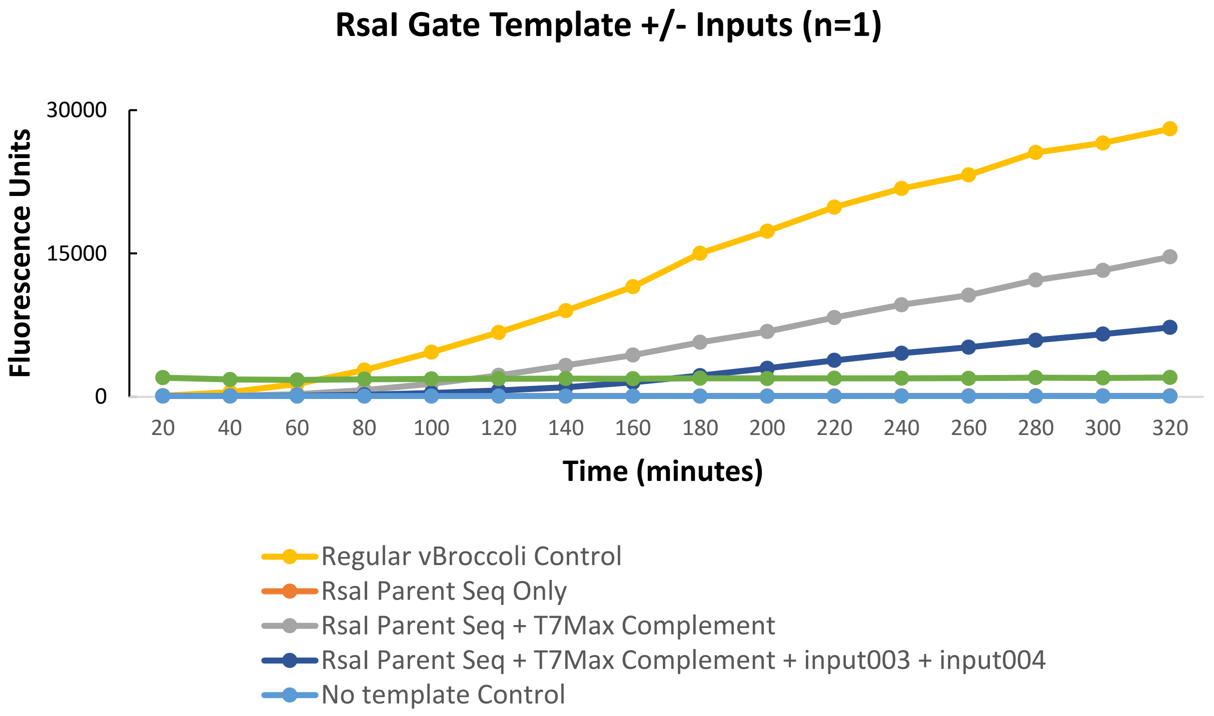
RsaI Gate Template +/- Inputs (n=1)
Fluorescence Units
30000
15000
0
20 40 60 80 100 120 140 160 180 200 220 240 260 280 300 320
Time (minutes)
Regular vBroccoli Control
RsaI Parent Seq Only
RsaI Parent Seq + T7Max Complement
RsaI Parent Seq + T7Max Complement + input003 + input004
No template Control

SI Figure 3: NAND gate example sequence designed on Benchling. The brown, unlabeled sequence is the PvuII recognition sequence.

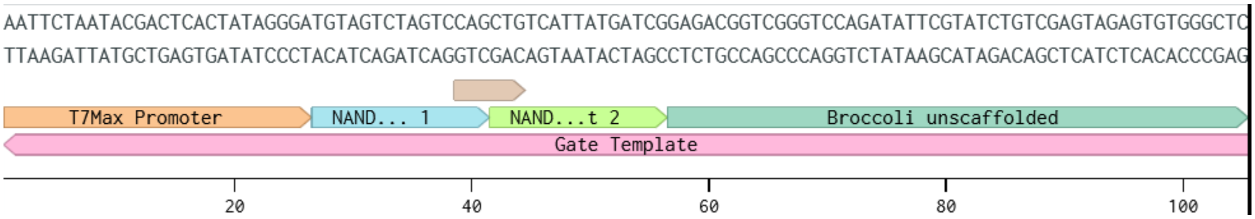

SI Figure 4: An mFold (a) and NuPack (b) depiction of the predicted secondary fold of Broccoli, an RNA aptamer. This specific secondary structure is necessary so that Broccoli can bind and activate the fluorescence of the ligand DFHBI (4-[(3,5-difluoro-4-hydroxyphenyl)methylidene]-1,2-dimethyl-4,5-dihydro-1H-imidazol-5-one). (c) The mFold depiction is a prediction of how we want Broccoli to fold when the RNA aptamer sequence is downstream of the gate sequence.

a.

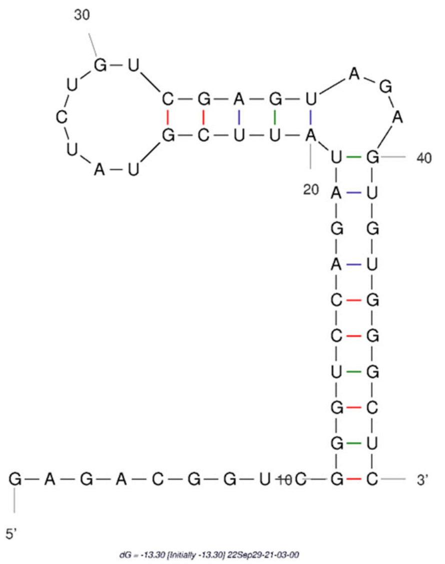

b.

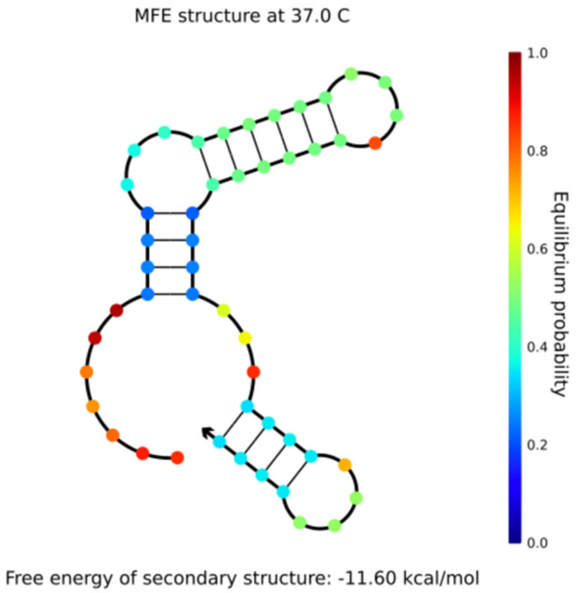

c.

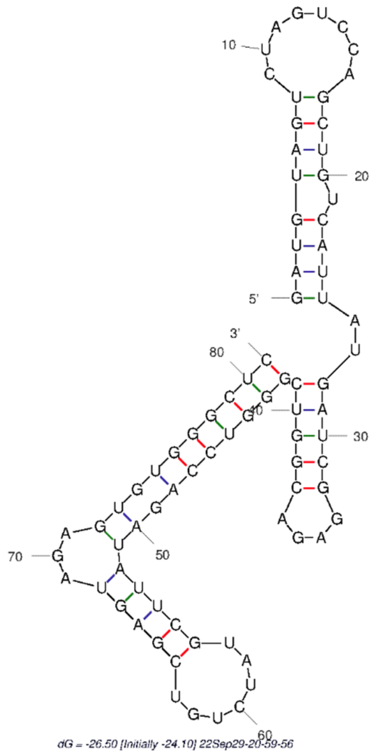

SI figure 5: Input concentration presented as differences in ratio of gate template concentration to input concentration. The ratio “1 to 3” was found to be the most effective for restriction enzyme digests through Urea PAGE analysis. However, fluorescence of transcribed RNA aptamers shows that the differences are negligible in contrast to samples where no inputs are provided.

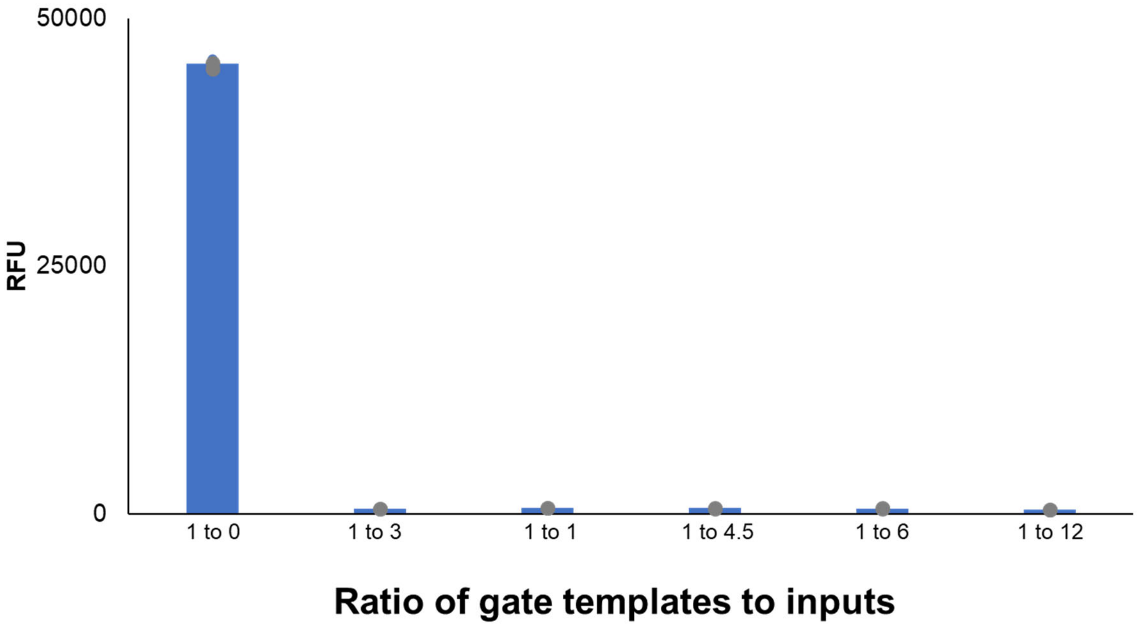

SI Figure 6: Variations in enzyme concentration where 10U of PvuII is the “normal” amount used for most experiments in this study.

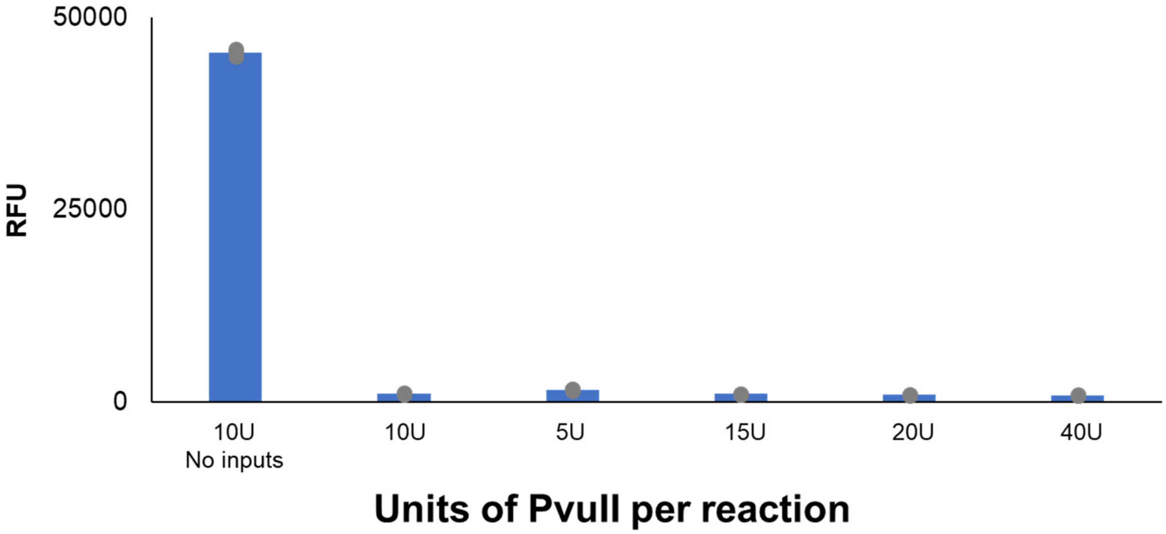

SI Figure 7: Decreasing concentrations of both gate templates and inputs to test the lower limits of gate function and cell free transcription. (a) The first concentration is the typical concentration used in most of the single gate experiments in this study; gate templates are 2µM and inputs are 6µM. “No inputs” indicate samples where the gate template and T7 Max sense complementary sequence were present, but the corresponding inputs were not. The next set of concentrations are gate templates at 1µM and inputs are 3uM, gate templates at 0.5µM and inputs at 1.5µM. Finally, the last gate template gate template concentrations in this graph were 0.1µM or 100nM and input concentrations were 0.3µM or 300nM. Although the SEM values for the 100nM gate template samples do show statistical difference and are detectable with fluorescence, the 2uM and 1uM samples are far more easily detectable so these were often the default concentrations used in the study. (b) The visual appearance of the samples when they were placed over a UV Transilluminator. (c) Gate template and input concentrations can be further decreased all the way down to 10nM. Although the global levels of potential fluorescence decrease as the concentration of the gates and inputs decrease, we can still see a difference in fluorescence between the 1 and 0 signals when using 10nM of gate templates.

a.

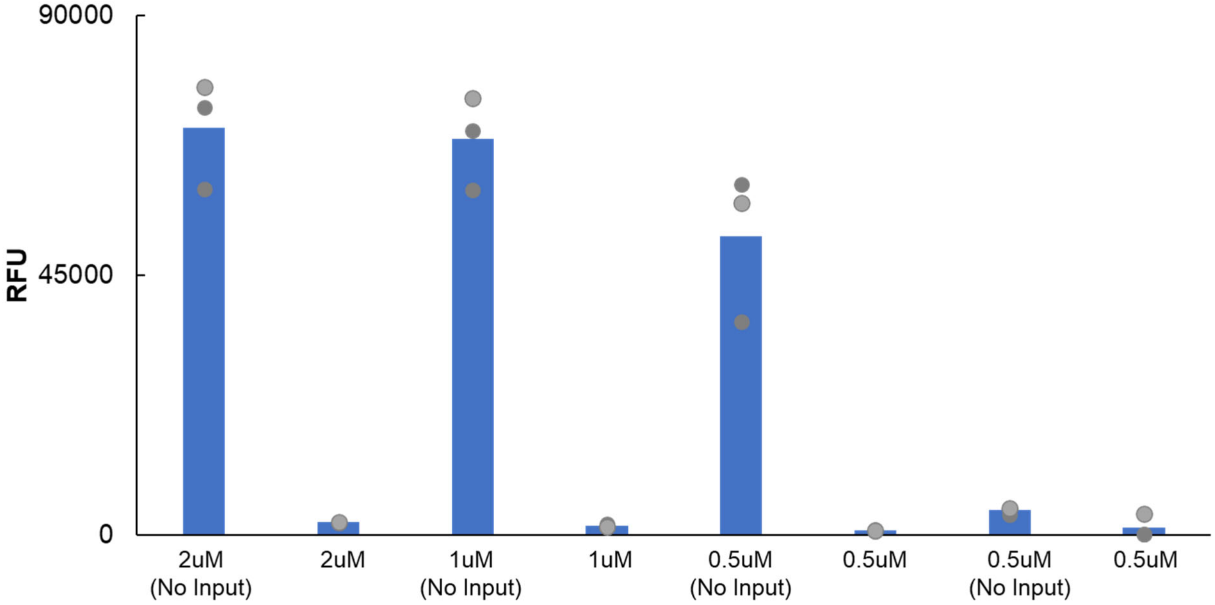

b.

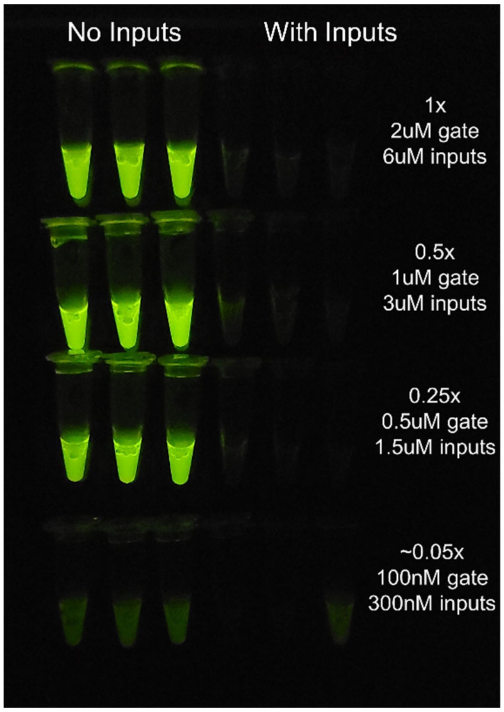
No Inputs
With Inputs
1x
2uM gate
6uM inputs
0.5x
1uM gate
3uM inputs
0.25x
0.5uM gate
1.5uM inputs
~0.05x
100nM gate
300nM inputs

c.

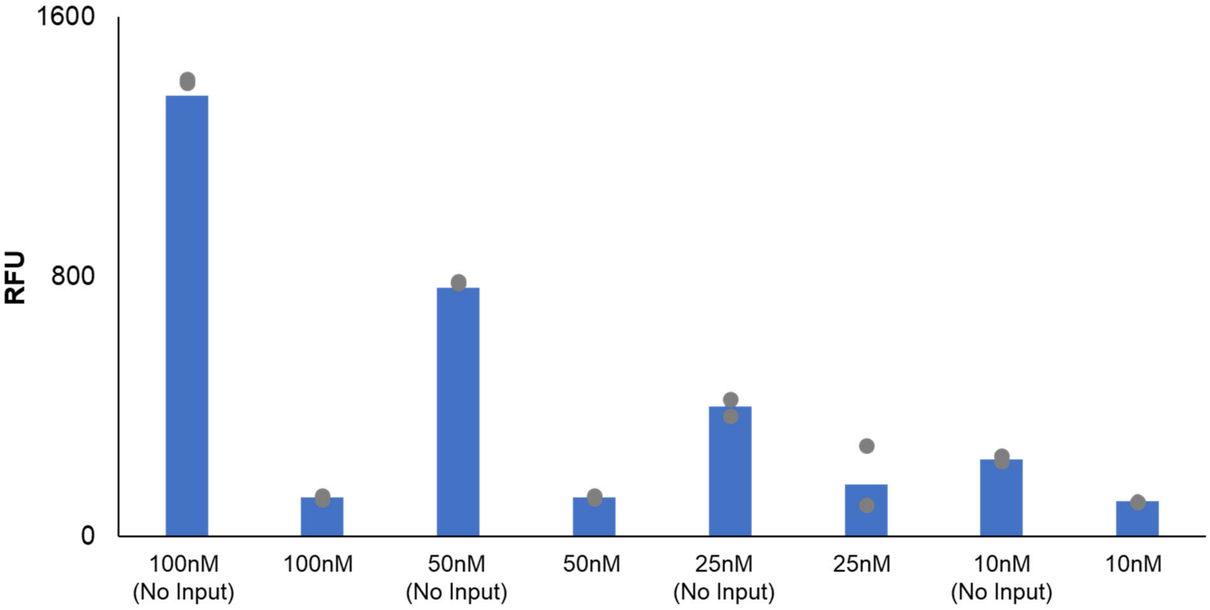
1600
800
0
RFU
100nM
(No Input)
100nM
50nM
(No Input)
50nM
25nM
(No Input)
25nM
10nM
(No Input)
10nM

SI Figure 8: NOT gate example designed on Benchling. The brown, unlabeled sequence is the PvuII recognition sequence.

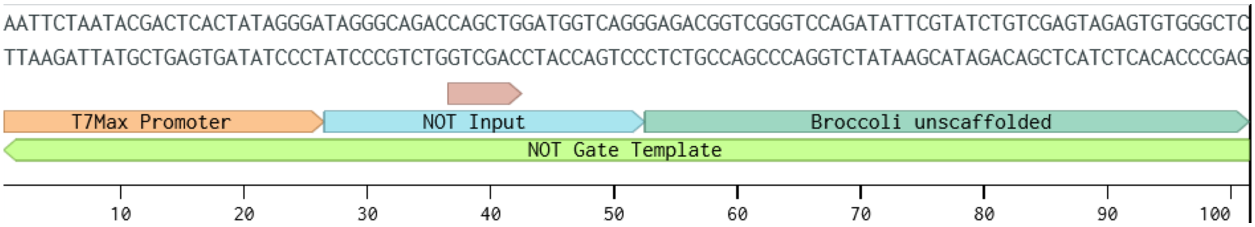

SI Figure 9: NOR gate example designed on Benchling. The brown, unlabeled sequence is the PvuII recognition sequence.

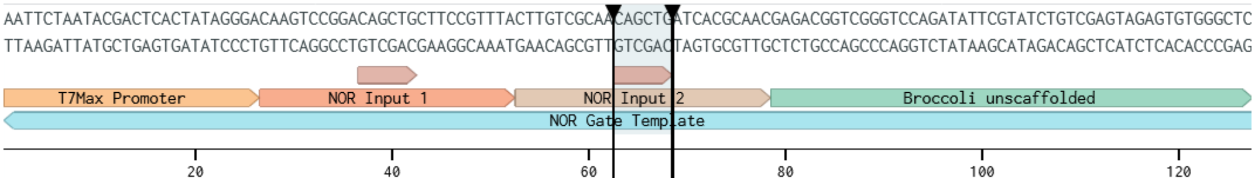

SI Figure 10: AND gate example designed on Benchling. The brown, unlabeled sequence is the PvuII recognition sequence.

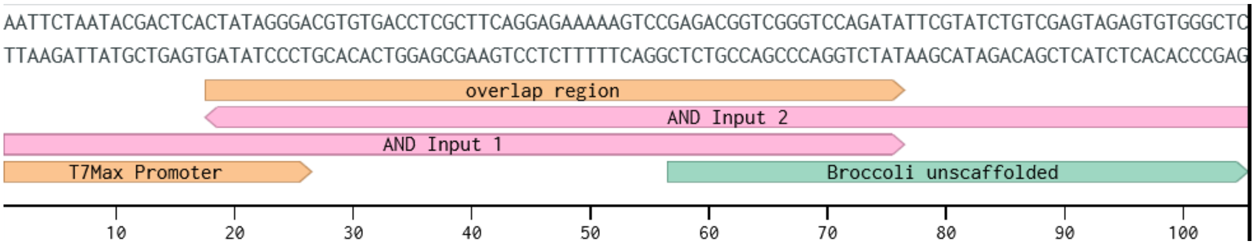

SI Figure 11: OR gate example designed on Benchling. The brown, unlabeled sequence is the PvuII recognition sequence.

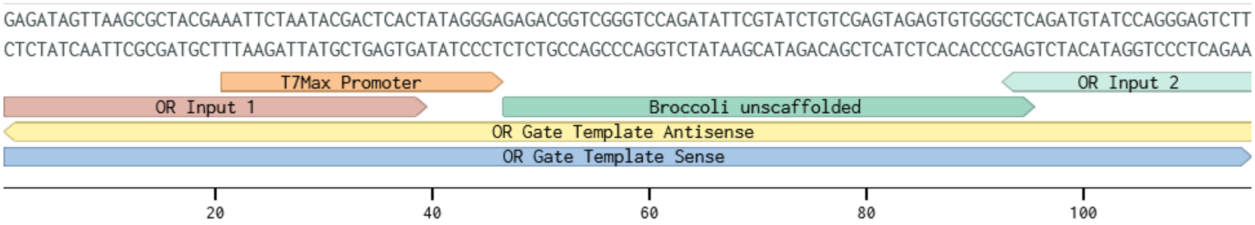

SI Figure 12: NAND Gate 1 of the multigate OR processor. Oligo 3885 is the antisense gate template. Oligo 3914 is the sense complement to the T7 Max promoter on the gate template. This oligo is conjugated to a biotin molecule on the 5' end. Oligo 2786 is Input 1A and oligo 2787 is Input 1B. Oligo 3886 is the sense release oligo. Oligo 3263 is the released oligo that becomes input 3A for NAND Gate 3.

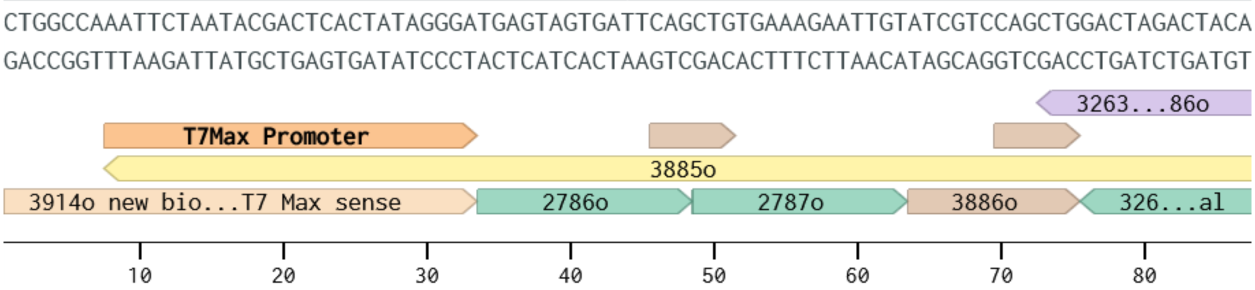

SI Figure 13: NAND Gate 2 of the multigate OR processor. Oligo 4051 is the antisense gate template. Oligo 3914 is the sense complement to the T7 Max promoter on the gate template. This oligo is conjugated to a biotin molecule on the 5' end. Oligo 4051 is Input 2A and oligo 4052 is Input 2B. Oligo 3886 is the sense release oligo. Oligo 3264 is the released oligo that becomes input 3B for NAND Gate 3.

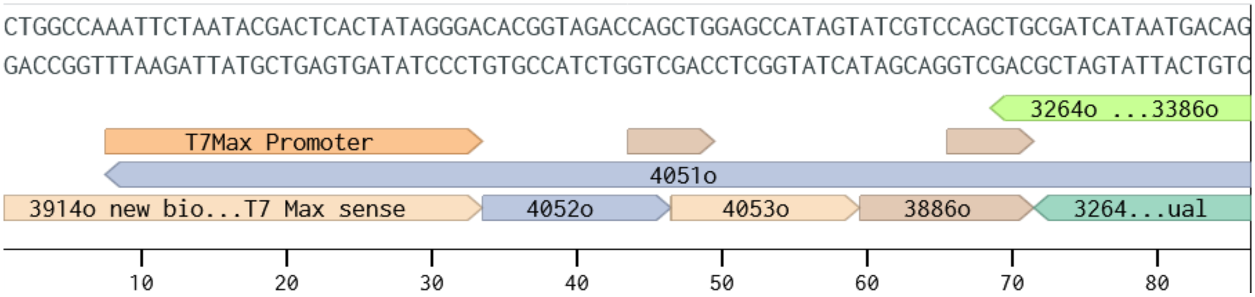

SI Figure 14: NAND Gate 3 of the multigate OR processor. Oligo 3386 is the antisense gate template. Oligo 3263 is Input 3A (output from NAND Gate 1). Oligo 3264 is Input 3B (output from NAND Gate 2). Oligo 2782 is the non-biotinylated T7 Max promoter sense complement.

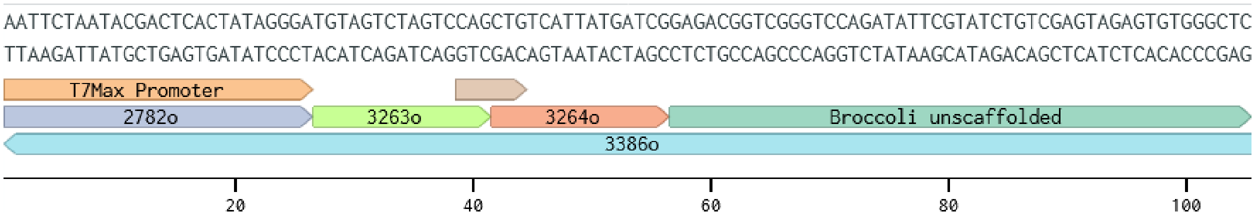

Supplement: Supplementary file 1 — Supplementary Information [file 41467_2023_37752_MOESM1_ESM.pdf]
