# Supplementary material for: Trumpet is an operating system for simple and robust cell-free biocomputing
Source: Nat Commun. 2023 Apr 20;14:2257. doi: 10.1038/s41467-023-37752-x (PMC10119096; doi:10.1038/s41467-023-37752-x)
Supplement: Supplementary file 8 — Description of Additional Supplementary Files [file 41467_2023_37752_MOESM8_ESM.pdf]

Title: Supplementary Data 1

Description: Initial digestion experiment testing functionality of restriction enzymes in version 1 of aHOT 7.9 buffer ("Homemade OneTaq 5x"). All data can be found in spreadsheet "SI1_Initial NAND Data". Information in the individual tabs within the spreadsheet are as follows:

**Tab**	**Information**
1001	New England BioLabs restriction enzymes list narrowed down according to functionality in OneTaq buffer, no heat inactivation, and no ambiguity of bases in recognition sites
1002	Gel Data Legend
1003	Digestion testing of PvuII, BamHI, StuI, Mfe in various buffers
1004	Digestion testing of BsaAI, BstUI, HpaI, NruI in various buffers
1005	Digestion testing of MfeI retest in various buffers
1006	Digestion testing of PaeR71, RsaI, ApaLI in various buffers
1007	Digestion testing of AvrII in various buffers

Title: Supplementary Data 2

Description: All individual gate data from gates designed for orthogonality experiments can be found in separate SI spreadsheet labeled "SI9_Orthogonality Data". Information in the individual tabs within the spreadsheet are as follows:

Tab	Information
1008	Cell free transcription of broccoli fluorescence data over 5 hours for **Gate Templates A** (internal ID 3386) **and B** (internal ID 3387) from Figure 1m heatmap.
1009	Cell free transcription of broccoli fluorescence data over 5 hours for **Gate Template C** (internal ID 3388) from Figure 1m heatmap.
1010	Cell free transcription of broccoli fluorescence data over 5 hours for gate template (internal ID 3389) **not included** in Figure 1m heatmap data and **Gate Template D** (internal ID 3390).
1011	Cell free transcription of broccoli fluorescence data over 5 hours for (internal ID 3391) **not included** and **Gate Template E** (internal ID 3392) from Figure 1m heatmap.
1012	Cell free transcription of broccoli fluorescence data over 5 hours for gate templates (internal IDs 3393) **not included** and **Gate Template F** (internal ID 3394) in Figure 1m heatmap data.
1013	Cell free transcription of broccoli fluorescence data over 5 hours for gate templates (internal ID 3395) **not included** in Figure 1m heatmap data.
1014	Cell free transcription of broccoli fluorescence data over 5 hours for **Gate Templates G** (internal ID 3396) and (internal ID 3397) **not included** from Figure 1m heatmap.
1015	Cell free transcription of broccoli fluorescence data over 5 hours for **Gate Template H** (internal IDs 3398) and (internal ID 3399) **not included** in Figure 1m heatmap data.
1016	Cell free transcription of broccoli fluorescence data over 5 hours for gate template (internal ID 3400) **not included** from Figure 1m heatmap.
1017	Cell free transcription of broccoli fluorescence data over 5 hours for gate template (internal ID 3401) **not included** in Figure 1m heatmap data.

Title: Supplementary Data 3

Description: Sequence-specific information can be found in separate document labeled "SI17_Gate Sequences". Information in the individual tabs within the spreadsheet are as follows:

**Tab**	**Information**
1018	Miscellaneous sequences
1019	NAND gate template and input sequences
1020	NOT gate template and input sequences
1021	NOR gate template and input sequences
1022	AND gate template and input sequences
1023	OR gate template and input sequences

Title: Supplementary Software
Description: The Python script code for Trumpet website.
